# Intensive care unit tracheostomy: a snapshot of UK practice

**DOI:** 10.1186/1755-7682-1-21

**Published:** 2008-10-25

**Authors:** Tonny Veenith, Sangeetha Ganeshamoorthy, Thomas Standley, Joseph Carter, Peter Young

**Affiliations:** 1 Department of Anaesthetics, Box 93, Addenbrooke's Hospital, Hills Rd, Cambridge, CB2 20QQ, UK; 2 Department of Anaesthetics, Queen Elizabeth Hospital, Gayton Rd, Kings Lynn, Norfolk, PE30 4ET, UK

## Abstract

**Background and methods:**

Tracheostomy is a common procedure in intensive care patient management. The aim of this study was to capture the practice of tracheostomy in Intensive Care Units in the United Kingdom. A postal survey was sent to the lead clinicians of 228 general intensive care units (ICUs) throughout the United Kingdom excluding specialist units. We aimed to identify the current practice of tracheostomy, including timing of insertion, equipment used and post-operative care and follow-up.

**Results:**

A response rate of 86.84% was achieved. Percutaneous tracheostomy continues to be favoured over surgical tracheostomy with less than 8% of ICUs opting for surgical tracheostomies > 50% of the time. 89% of units required only 2 operators to perform the technique and single stage dilatation is the technique of choice in 83% of units. The Ciaglia technique, which was strongly favoured less than a decade ago, is currently practiced in less than 5% of ICUs. Bronchoscopic guidance is an important adjunct to the technique of percutaneous tracheostomy with 80% of units using it routinely. Follow-up care of patients remains poor with 59% of ICUs not having routine follow-up once the patient has left the unit.

**Conclusion:**

The practice of percutaneous tracheostomy remains the preferred technique within the UK. There seems to be a growing preference for single stage dilatational techniques. Timing of tracheostomy remains variable despite evidence to suggest benefit from an earlier procedure. Follow-up of tracheostomised patients after discharge from ICU is still low, which may mean significant morbidity from the procedure is being missed.

## Background

The cost of hospital care is under increasing financial pressure in the developed world, with some estimates placing the cost of critical care services alone at approximately 1% of a nation's Gross Domestic Product (GDP) [1]. One of the commonest management strategies in intensive care units is mechanical ventilation and tracheostomy in intensive care is usually performed for patients requiring prolonged mechanical ventilation [2]. Over the last 10 years the number of tracheostomies performed has increased rapidly and tracheostomy has been shown to reduce the duration of ICU stay [3].

Shelden (1957) first introduced percutaneous tracheostomy many years after the technique of modern surgical tracheostomy was described by Jackson in 1909 [4,5]. The percutaneous technique was further refined by Ciaglia (a general thoracic surgeon) in 1985 [6]. The practice of ICU tracheostomy has constantly been evolving since, with the introduction of new equipment as well as the widespread use of fibre-optic bronchoscopic technology. Percutaneous tracheostomy is currently regarded as a cost-effective, safe alternative to the open surgical technique and carries the advantage of being widely practised as a bedside procedure [7].

Two major meta-analyses comparing surgical versus percutaneous tracheostomy (both limited by heterogeneity) arrived at different conclusions as to which approach was superior. Dulguerov et al [8] concluded that percutaneous tracheostomies were inferior their surgical equivalent whereas Freeman et al [9] found percutaneous tracheostomies easier to perform with fewer associated complications. Many techniques exist for performing percutaneous tracheostomy, the previous preferred practice being serial dilatation which was further simplified by a two stage dilatation technique [6,10]. The work of Polderman et al suggests that percutaneous tracheostomy with a kit (Portex; Hythe, Kent, UK) with curved dilating forceps and bronchoscopic guidance is as effective as surgical tracheostomy [11,12].

However, uncertainty still surrounds tracheostomy as an ICU procedure. There is doubt as to the benefit of tracheostomy for prolonged mechanical ventilation in unselected patients and the issue of timing remains contentious. Clec'h et al suggests that tracheostomy does not reduce intensive care unit mortality when performed in unselected patients and may represent a burden after intensive care unit discharge [13]. However, good quality prospective studies by Rumback and Moller show that tracheostomy reduces the incidence of ventilator associated pneumonia in intensive care [11,14]. There have been valuable surveys in the past evaluating United Kingdom (UK) ICU tracheostomy practice [15-17]. The aim of this study was to capture the current practice of tracheostomy in the UK in the context of current thinking about the procedure and previously documented practice.

## Materials and methods

A questionnaire (see additional file 1) was developed in our ICU and sent to the lead clinicians in all general intensive care units across the UK, with a covering letter and a prepaid envelope. This survey specifically aimed to identify variations in current tracheostomy practice including timing of insertion, equipment used, post-operative care and how patients were followed up. For the purpose of comparing with previous surveys all general ICUs across the UK were sent questionnaires (addresses sourced from The Directory of Critical Care 2003' – CMA Medical Data, Loughborough, UK). All specialist ICUs (paediatric, neurosurgical, cardiothoracic and liver units) were excluded like previous surveys, to allow a fair comparison.

## Results

From the 228 units we posted the questionnaire to, we received a prompt response from 197 units, achieving a response rate of 86.84%. Of the 197 responders, 3 units did not have a general intensive care unit, and were therefore excluded. 56% of units carried out less than 50 tracheostomies per year with only 4.1% performing more than 200.

### Timing of tracheostomy in the UK

**Figure 1:**
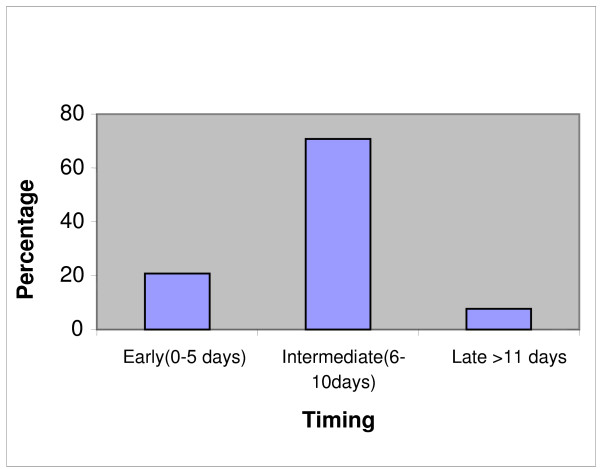
Timing of UK ICU tracheostomy.

Our survey revealed that 21% of responders carry out tracheostomy early (between 0 – 5 days), 71% between 6 to 10 days, and 8% of the units > 10 days (see Figure 1).

### Method of insertion

#### Surgical versus percutaneous tracheostomy

Percutaneous tracheostomy is preferred over the surgical technique. In 43% of units, tracheostomies are performed percutaneously > 95% of the time. In 32.4% of units, 75–95% of the tracheostomies are carried out via the percutaneous route and 16.6% of units perform between 50 to 75% of their tracheostomies percutaneously. 8% of units prefer the surgical technique performing fewer than 50% of their tracheostomies percutaneously.

#### Number of doctors required for the procedure

The majority of units (89.4%) performed the procedure with two doctors, 8.3% of units performed the procedure with 3 doctors, and the remaining 2.3% carried out the procedure with a single doctor. All the hospitals practicing a one-doctor tracheostomy technique were performing less than 60 tracheostomies per year.

#### Sterility

All units observed sterile hand wash precautions and wore sterile gloves during tracheostomy insertion. The majority of operators (98%) wore sterile gowns; masks by 60%. Only 1% of units failed to use any sterile drapes during the insertion of percutaneous tracheostomies.

#### Bronchoscopic guidance during tracheostomy

Most units (80%) perform all percutaneous tracheostomies under bronchoscopic guidance whereas 20% don't use bronchoscopic guidance routinely. Of the latter 20%, 10% of units use the bronchoscope if a difficult tracheostomy is anticipated. 7% use more bronchoscope more than 50% time and 3% of units never utilise a bronchoscope during insertion of a percutaneous tracheostomy; the final category do more than 100 tracheostomies per year.

#### Maintenance of airway during percutaneous tracheostomy

Of the respondents 92% of units used the original cuffed endotracheal tube for the maintenance of the airway on insertion of a percutaneous tracheostomy. The vast majority of units (168) pull the original endotracheal tube back, and a minority (7 units) pushed the endotracheal tube further into the trachea during the procedure. Other infrequently used airways in addition to endotracheal tubes were supraglottic airways (7%) and a microlaryngeal tube (2%). Units where microlaryngeal tubes were used for airway maintenance carried out the procedure without bronchoscopic guidance.

#### Use of vasoconstrictors in local anaesthetics for insertion

Local anaesthetic with adrenaline was a common choice as the local anaesthetic during the procedure (95%). Only a minority used local anaesthetic without a vasoconstrictor (5%).

#### Favoured tracheostomy technique

**Figure 2:**
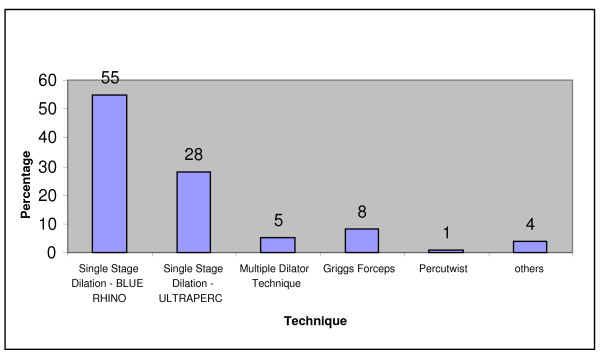
Favoured tracheostomy technique.

Single stage dilatation was commonly used to perform tracheostomy. The favoured techniques were Blue Rhino (55%) and Ultraperc (28%). Griggs forceps (8%) and multiple dilator technique (5%) were used occasionally. 4% of the intensive care units used more than one technique for their percutaneous tracheostomies (Figure 2).

### Post insertion care

#### Normal frequency of tracheostomy tube change

A quarter of the units changed their tubes routinely within 14 days; another quarter changed it routinely within 28 days. Half of the units did not change their tracheostomy tube before 28 days or changed only when the tube was blocked.

#### Use of inner liners

More than half (51%) of the intensive care units routinely used inner liners for the tracheostomy tubes. 31% of them used inner liners occasionally whereas 18% never used them at all.

### Decannulation and follow-up

Decannulation is performed mainly by the intensive care nurses in 43% of units, by a combination of clinicians in 31%, doctors alone in 23%, and physiotherapist alone in 2%.

Follow-up was performed mainly by ICU review clinics (29%) and ENT and Outreach teams (12%). However, 59% of patients who had an ICU tracheostomy did not receive any follow up once they had left the unit.

## Discussion

In our survey percutaneous tracheostomy is the widely used ICU tracheostomy method, which coincides well with the trend in current literature [15-18]. The percutaneous technique seems to have gained universal acceptance in the hands of intensivists. There is wide variation in the complication rates following percutaneous tracheostomy; ranging from 7 – 19% [18,19]. The randomised controlled trial by Silvester et al demonstrated no significant difference in complication rate between percutaneous dilatational and surgical tracheostomy, on long term follow-up to 20 months, albeit for a higher incidence of infection at day 7 following surgical tracheostomy [19]. The fact that complication rates for the percutaneous technique in the hands of doctors whose primary training is often not surgical, is surely a major factor in the generalised acceptance of the technique demonstrated in this and other surveys.

**Figure 3:**
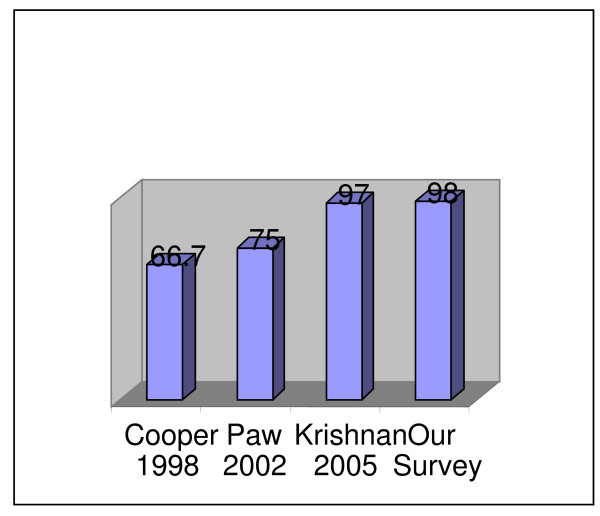
Progression of percutaneous tracheostomy (numbers in%).

Taking a historical view with the other notable surveys of UK ICU tracheostomy in the last decade, there is a tangible increase in the number of hospitals providing a percutaneous tracheostomy service [15-17] (Figure 3).

This increase may also be attributed to increasing experience and confidence with the percutaneous technique in addition to the widespread provision of fibre-optic bronchoscopes. Fibre-optic bronchoscopy is now much more accepted as an adjunct for percutaneous tracheostomy. Over a decade ago in the survey by Paw et al [15] only 49% of ICUs used routine fibre-optic bronchoscopy for percutaneous tracheostomy. In our survey over 80% of ICUs use routine fibre-optic bronchoscopy. The preference for a single stage dilatation technique is likely due to ease of use and decreased operative times compared to the serial-dilatation technique, without the risk of increased complications [20].

### Timing

Although tracheostomies are routinely performed in intensive care units there is yet no clear evidence regarding the optimal time of insertion for this technique. Rumback et al demonstrated in a sound prospective double blind controlled trial that there is significant reduction in ICU mortality and length of stay after an early tracheostomy [11]. The TracMan trial run by the Intensive Care Society of the UK has recruited 788 patients at the time of preparation of this manuscript and when completed may give a more definitive answer to the timing of tracheostomy in UK ICU's [21].

Our survey demonstrates mixed practice with regard to the timing of ICU tracheostomy. Whilst, early tracheostomy undoubtedly benefits some patients, too proactive an approach to tracheostomy based purely on anticipated length of ventilation may not be in every patient's best interest, particularly where underlying cardiorespiratory physiological function is good and the underlying condition that has resulted in ICU admission is readily reversible. We did not take any specific details of the casemix of the units surveyed – to have done would have increased the data collected enormously – and so, cannot make any comment as to the appropriateness of the mixed practice seen. Nonetheless, it is likely that opinions on this issue do vary.

### Post tracheostomy care and follow-up

Routine post insertion change of the tracheostomy tube every two weeks helps to reduce granulation tissue, incidence of tracheal stenosis and bacterial contamination of the stoma [22]. In our survey half the units changed the tube after 28 days or only when it was blocked. Perhaps more alarming is the lack of follow up to identify longer term complications following ICU tracheostomy. Compared to previous surveys [15-17], follow-up after ICU discharge has marginally increased to 41%. Lack of solid data on the consequences of ICU tracheostomy is surely needed on each and every unit in order to allow a fuller risk/benefit assessment for each patient where tracheostomy is being considered, especially if significant morbidity is being missed from the procedure. Knowledge of the complications that occur may also help to inform which patients should receive a tracheostomy and when.

## Conclusion

This survey has created a snapshot of the current practice of ICU tracheostomy in the UK. Percutaneous tracheostomy is the preferred technique in the majority of units when compared to surgical tracheostomy, with a single dilatation technique being favoured by the majority. There is mixed practice with regards to the timing of ICU tracheostomy, which suggests heterogeneous opinions. There is still a fairly low level of routine follow-up after ICU discharge, which may mean significant morbidity is being missed, representing a major omission in our attempt to fully understand the risks and benefits to individual ICU patients.

## Competing interests

The authors declare that they have no competing interests.

## Authors' contributions

TV, SG, PY conceived the study, and participated in its design and coordination. JC, TS revised the manuscript after peer review with full access to survey data. All authors read and approved the final manuscript.

## Supplementary Material

Additional file 1 **Survey of Percutaneous Tracheostomies in ICU.** Click here for file
