# Supplementary material for: Intensive care unit tracheostomy: a snapshot of UK practice
Source: Int Arch Med. 2008 Oct 25;1:21. doi: 10.1186/1755-7682-1-21 (PMC2583967; doi:10.1186/1755-7682-1-21)
Supplement: Additional file 1 — Survey of Percutaneous Tracheostomies in ICU. [file 1755-7682-1-21-S1.doc]

# *Survey of Percutaneous Tracheostomies in ICU*

*Name of ICU:*

# *Please tick as appropriate*

| Approximate number of tracheostomies performed in your ICU annually | ------------------------- |
| --- | --- |
| Preferred method of elective tracheostomy for your ICU? | - Surgical - Percutaneous |
| Normal number of doctors required for the procedure? | - 1 - 2 - 3 |
| Approximately what percentage of tracheostomies are done percutaneously (as opposed to surgically) in your ICU? | ---------------------------- |
| What percentage of percutaneuous tracheostomies are done under bronchoscopic guidance in your ICU? | - 100 - 50 - 100 - < 50 - None |
| Normal timing of tracheostomy in your ICU? | - Early 0 – 5 days - Intermediate 6 – 10 days - Late 11+ days |
| Which sterile techniques do you normally utilise on your ICU?  (tick all that apply) | - Hand wash - Gloves - Gown - Mask - Drapes |
| Do you infiltrate with a vasoconstrictor prior to Percutaneous tracheostomy on your ICU | - Yes - No |
| Normal airway maintenance during the procedure? | - Original ETT pushed in - Original ETT pulled back - Microlaryngeal tube - Supraglottic airway |
| Favoured tracheostomy technique? | - Single stage dilation (Blue Rhino) - Single stage dilation (UltraPerc) - Multiple dilator techniques - Griggs forceps - PercuTwist - Other-if so, please state below   _______________________ |
| Normal frequency of routine tracheostomy tube change on your ICU? | - 0 – 14 days - 14 - 28 days - > 28 days - Only if blocked |
| Are disposable inner tubes utilised in your ICU? | - Normally - Occasionally - Never |
| Who performs the decannulation in your ICU? | - ICU nursing staff - ICU doctors - Physiotherapists |
| Any long-term follow-up offered? | - None - ICU Review clinic - ENT review - Other --------------------------------- |
